# Visual Search for Wines with a Triangle on the Label in a Virtual Store

**DOI:** 10.3389/fpsyg.2017.02173

**Published:** 2017-12-13

**Authors:** Hui Zhao, Fuxing Huang, Charles Spence, Xiaoang Wan

**Affiliations:** ^1^Department of Psychology, School of Social Sciences, Tsinghua University, Beijing, China; ^2^Crossmodal Research Laboratory, Department of Experimental Psychology, University of Oxford, Oxford, United Kingdom

**Keywords:** visual search, virtual reality, triangle, wine labels, DPTS effect

## Abstract

Two experiments were conducted in a virtual reality (VR) environment in order to investigate participants’ in-store visual search for bottles of wines displaying a prominent triangular shape on their label. The experimental task involved virtually moving along a wine aisle in a virtual supermarket while searching for the wine bottle on the shelf that had a different triangle on its label from the other bottles. The results of Experiment 1 revealed that the participants identified the bottle with a downward-pointing triangle on its label more rapidly than when looking for an upward-pointing triangle on the label instead. This finding replicates the downward-pointing triangle superiority (DPTS) effect, though the magnitude of this effect was more pronounced in the first as compared to the second half of the experiment, suggesting a modulating role of practice. The results of Experiment 2 revealed that the DPTS effect was also modulated by the location of the target on the shelf. Interestingly, however, the results of a follow-up survey demonstrate that the orientation of the triangle did not influence the participants’ evaluation of the wine bottles. Taken together, these findings reveal how in-store the attention of consumers might be influenced by the design elements in product packaging. These results therefore suggest that shopping in a virtual supermarket might offer a practical means of assessing the shelf standout of product packaging, which has important implications for food marketing.

## Introduction

Product packaging constitutes a powerful marketing tool (see Spence, 2016, for a recent review). It allows manufacturers and marketers to deliver important information regarding the product, attract consumers’ attention, and ultimately influence their evaluation of a product via various specific features, including color, shape, curvature, label, typeface, and so on (for comprehensive reviews, see Hine, 1995; Simmonds and Spence, 2017). As for food and drink purchases, consumers have their first sensory contact with the products via what they see. Visual cues also provide a dominant sense as far as the generation of product expectations are concerned (Schifferstein et al., 2013; Piqueras-Fiszman and Spence, 2015). Packaging not only affects how food and drinks are perceived at the point of sale (e.g., Becker et al., 2011; van Rompay et al., 2016), but can also influence how they are experienced at the point of consumption (Schifferstein et al., 2013; Spence, 2016).

Over the years, many studies have been designed to examine how the attention of consumers in-store is affected by the design elements in product packaging (e.g., Schoormans and Robben, 1997; Underwood et al., 2001; Pieters et al., 2010; Otterbring et al., 2013). In the store setting, of course, each and every product on the shelves competes for the consumer’s limited visual attention. Nevertheless, according to Hoyer (1984), it only takes a few seconds for a customer to make their decisions when buying fast-moving consumer goods (FMCGs). Perhaps unsurprisingly, it has been argued that the more attention a customer pays to a product, the greater the likelihood that they will choose it (see Orquin and Loose, 2013, for a comprehensive review). Compared to changing the appearance of the product itself, changing the packaging and labeling of the product may well constitute a more cost-efficient, but no less effective means of attracting the attention of customers.

Purchasing wine constitutes a particular challenge (relative to other product categories). In fact, searching for a specific brand/vintage in the wine aisle can be both difficult and challenging, given the often complex and ever-changing range on products one finds for sale on the wine shelves. The appearance of the bottles, especially the design of the wine labels, might be expected to influence a consumer’s liking of, or preference for, the product (Cutler, 2006; Boudreaux and Palmer, 2007; Labroo et al., 2008; De Mello and Pires, 2009; Westerman et al., 2013; Gmuer et al., 2015). What is more, it can also guide customers’ attention in searching for the product presented on the store shelves (Thomas and Pickering, 2003; Elliot and Barth, 2012).^1^

The visual search task has been one of the most common experimental paradigms used to study attention (e.g., Nothdurft, 1999; Hopf et al., 2000). In visual search, certain shapes are easier to find than others. For example, a circle with a straight line like a “Q” is easier to find amongst regular circles than vice versa (Treisman and Souther, 1985; Treisman and Gormican, 1988). Such research show that it is easier to search for the presence of a feature than for its absence. Searching for a downward-pointing triangle among upward-pointing distractor triangles has also been shown to be faster than vice versa (Larson et al., 2007). The latter effect is known as the Downward-Pointing Triangle Superiority (DPTS) effect. The existence of this effect has been attributed to the fact that downward-pointing triangles are more likely to convey threat-related information than are upward-pointing triangles. It has been suggested that this is because they resemble angry faces in which the muscles are pulling down to form a “V” shape (Larson et al., 2012; Toet and Tak, 2013), and therefore capture attention more readily than do neutral stimuli such as upward-pointing triangles (see Larson et al., 2007, 2012; Watson et al., 2012).

It is widely acknowledged that the rapid detection of threat-related stimuli conveys an evolutionary advantage and is thus vital to human survival. The “Shape of Threat” account of the DPTS effect is also supported by neuroscience evidence showing that viewing downward-pointing triangles increases the neural response seen in the amygdala (Larson et al., 2009). This neural structure plays a crucial role in emotional processing, especially in the detection of potential threats (e.g., Morris et al., 1996; LeDoux, 2000).

It is important to note, however, that this threat-related explanation of the DPTS effect might not easily be used to account for the comparable pattern of results documented with non-threatening images of triangular-shaped foods and pizza packaging (Shen et al., 2015). Considering the global precedence in visual perception (e.g., Navon, 1977; Pomerantz, 1983), one possibility is that the global outline shape of the stimuli (i.e., the downward-pointing triangle which could be threat-related) might be processed before the meaning of the stimuli (food or food packaging which are not threat-related). Nevertheless, the DPTS effect has also emerged with wine bottles that have triangular shapes on their labels whereby the triangles were only local features (Zhao et al., 2016). What is more, compared to foods presented in triangular form, or food packaging which is often stacked horizontally, the wine labels attached to the front of wine bottles and typically presented vertically. This means that the triangles appearing on the wine labels may actually point downward or upward in both daily life and experimental research (see Shen et al., 2015, for an example of a downward-pointing triangle actually being used in a commercially successful wine label). By contrast, foods or food packaging in triangular form usually point toward or away from the observer in daily life, but they were presented vertically in Shen et al.’s (2015) experiments. Nevertheless, it remains unclear whether a similar DPTS effect would also emerge with consumers in a store setting. Here it is important to note that conducting field studies (e.g., in a supermarket) can be both expensive and time-consuming. By contrast, using virtual reality (VR) to simulate a store experience offers a potentially efficient and effective means of keeping the costs of research down and can be argued to increase the external validity of one’s research relative to other computer-monitor based lab experiments.

A computer-mediated three-dimensional environment can be created in VR in which users can experience a sense of presence, and which provides realistic visual scenes, sense of movement, and so on (e.g., Biocca, 1992; Steuer, 1992; though see also Gallace et al., 2012). In particular, VR can simulate naturalistic environments via an immersive human–computer interaction (Berneburg, 2007; Lee and Chung, 2008; Bressoud, 2013). It can also be used to efficiently deliver realistic food cues to elicit craving (at least as indexed by self-report and salivation) just like actual food does (Ledoux et al., 2013; though see also Spence, 2011). Intriguingly, virtual stores can elicit similar consumer choice of food products such as milk and cookies as those that have been documented in a physical store (van Herpen et al., 2016; see also Waterlander et al., 2015), and have been used to examine purchases of alcoholic drinks, such as beer (Bigné et al., 2016).

In the present study, we wanted to examine whether the DPTS effect would emerge in the visual search for bottles of wine in a virtual supermarket. Specifically, three questions were addressed by the research reported here: First, is the visual search for a wine bottle with a downward-pointing triangle on its label in a virtual supermarket significantly more efficient (that is, faster and/or more accurate) than when the target bottle had a triangle point upward? Second, considering that products presented on the top shelf typically receive more attention than those placed lower down (e.g., Chandon et al., 2009; though, see van Herpen et al., 2016, for the opposite pattern in consumers’ choice of milk and cookies), we also examined whether the DPTS effect in the visual search for wines might be modulated by the position of the target bottle on the store shelf. Third, how does the orientation of the triangular shape on the wine label influence people’s rating of the bottles themselves, and is their preference for the item related to the efficiency of their visual search performance for it?

## Experiment 1

Experiment 1 attempted to replicate Zhao et al.’s (2016) recent results using pictures of wine shelves in a virtual supermarket. The participants were invited to come to a psychology laboratory that was equipped with a head-mounted display (HMD) VR system, and to immerse themselves in a virtual supermarket to perform the visual search in a wine aisle.

### Methods

#### Participants

Twenty-four Chinese participants (mean age = 21.08 ± 1.95 years, ranging from 18 to 27 years; 12 females and 12 males) were recruited from the subject pool of the Applied Cognition Laboratory of Tsinghua University, Beijing, China.^2^ All of the participants reported having normal or corrected-to-normal vision. Each participant was compensated with 35 Chinese Yuan (CNY) for taking part in the study. The present study was approved by the ethics committee of the Psychology Department of Tsinghua University, and conformed to the ethical standards for conducting research established by the American Psychological Association.

#### Apparatus and Virtual Displays

**FIGURE 1:**
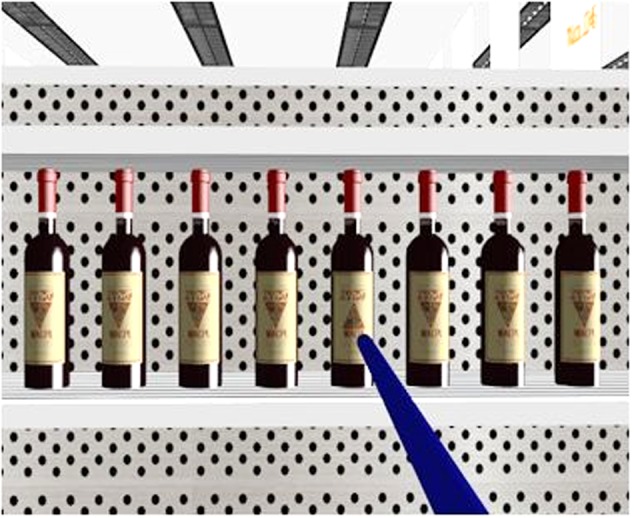
A screenshot from the left eye of the head-mounted display (HMD) for a search display (consisting of eight bottles of red wine as an example display) in Experiment 1. The blue rod was used to select the target.

Both of the experiments reported in the present study were conducted at the VR Laboratory at the Department of Psychology of Tsinghua University. An NVIS nVisor SX60 HMD VR system was used, which produces stereoscopic imagery at 60 frames per second per eye. The display for each eye had a resolution of 1280 (horizontal) × 1024 (vertical) pixels, and the optical field for each eye was 44° (horizontal) × 35° (vertical). **Figure 1** shows the screenshots from the left eye of the HMD. The participants used a Logitech F710 wireless gamepad to interact with the virtual environment, while the Vizard software (by WorldViz) was used to conduct the experiment and record the data.

The immersive virtual environment (IVE) in this study consisted of a supermarket created with the Autodesk 3Ds Max 2009 software. The whole supermarket was 20 m long, 15 m wide, and 5 m high, though the participants in the present study were only allowed to move along one of the aisles (7.6 m long and 1.6 m wide) with 4-shelf grayish white rack on both sides. There were a total of four racks on each side of the aisle, each of which consisted of four shelves with punch backboard. Each rack had a depth of 0.45 m. The base of the top, second, third, and bottom shelves were 1.77, 1.36, 0.94, and 0.5 m away from the ground, respectively. As can be seen in **Figure 1**, all of the bottles were presented on the top shelf. In order to make sure that all of the participants were able to see the same displays, the eye height in the virtual supermarket was set to 1.85 m regardless of the actual height of the participants. Therefore, when the participants looked at the rack, they were able to see all of the bottles (approximately 1.65 m away from where they were standing), without having to lift their heads.

**FIGURE 2:**
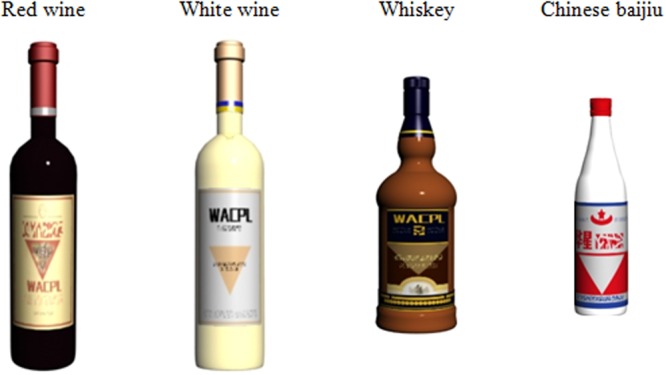
Four different bottles shown to participants in Experiments 1 and 2.

Within each display, there were a total of 8 bottles, 0.15 m apart from each other. As can be seen in **Figure 2**, the bottles were made of clear glass in order to reveal the color of the liquid within (approximately crimson, beige, brown, or clear), implying that the bottles contained red wine (0.41 m in height and 0.09 m in diameter), white wine (0.41 m in height and 0.09 m in diameter), whiskey (0.32 m in height and 0.11 m in diameter), or Chinese baijiu^3^ (0.27 m in height and 0.07 m in diameter), respectively. Each bottle had a downward- or upward-pointing triangle on its label, with fictional brand names. All of the bottles in each display presented the same wine, though one of the bottles was randomly determined to have a different label from the others. In other words, all of the trials in this experiment were the target-present trials.

In this virtual supermarket, the participants pressed a button on the gamepad in order to move along the aisle at a constant speed of 1.5 m/s, while keeping their bodies physically still. By contrast, they were instructed to physically turn their body around when needed. Thus, the information regarding transition was purely based on optic flow, whereas the information regarding rotation was based on both optic flow and body senses. Such a combination of cues has been shown to efficiently provide self-motion information to the participants (e.g., Wan et al., 2010, 2012).

#### Design and Procedure

The experimental task involved trying to find the bottle with a label that was different from the others, i.e., finding the one having a downward-pointing triangle on its label among others having upward-pointing triangles, or vice versa. After finishing a practice block of eight trials, each participant completed eight blocks of eight trials each. Each type of bottle was shown in 16 trials, with half with a downward-pointing target and an upward-pointing target in the remainder. All of the trials were mixed and presented in a random order. Within each trial, the location of the target was randomly determined.

At the beginning of each experimental block, the participants stood at one end of the aisle, adjacent to the rack on their left and facing the other end of the aisle. They were instructed to press a button on the gamepad to move forward along the aisle until a red arrow pointing to the right appeared in front of them, which instructed them to turn around to face the rack on their right where a display of eight bottles was presented. The red arrow disappeared when facing the display, and then a blue rod extending horizontally from their body to the display appeared (see **Figure 1** for an illustration). When the participants were searching for the target, they rotated their head to direct the blue rod to point to the target, and then pressed another button on the gamepad to pick it up. If they made a correct response, the target bottle disappeared; if not, a red cross appeared, and a short beep was played via the helmet they were wearing in order to alert them of the mistake. After that, a red arrow pointing to the left appeared to instruct them to turn left to face the aisle and then to proceed to the next trial. This procedure was repeated until the participants reached the end of the aisle where they were instructed to turn around to face the end of the aisle from which they had started and now had to perform the task with the displays on their left. When they arrived back at the start of the aisle, which means they had finished a total of eight trials, the block ended.

#### Data Recording

During each trial, the participant’s response (to select the target) was recorded. We used two dependent variables to assess the participants’ visual search performance, including: (1) the responses times (namely the RTs), consisting of the response latencies (from seeing the display to starting moving), the time durations to start moving to pointing the target, and the time durations to press a button on the game pad to confirm the selection, and (2) accuracy to indicate whether the target was correctly selected.

### Results and Discussion

**FIGURE 3:**
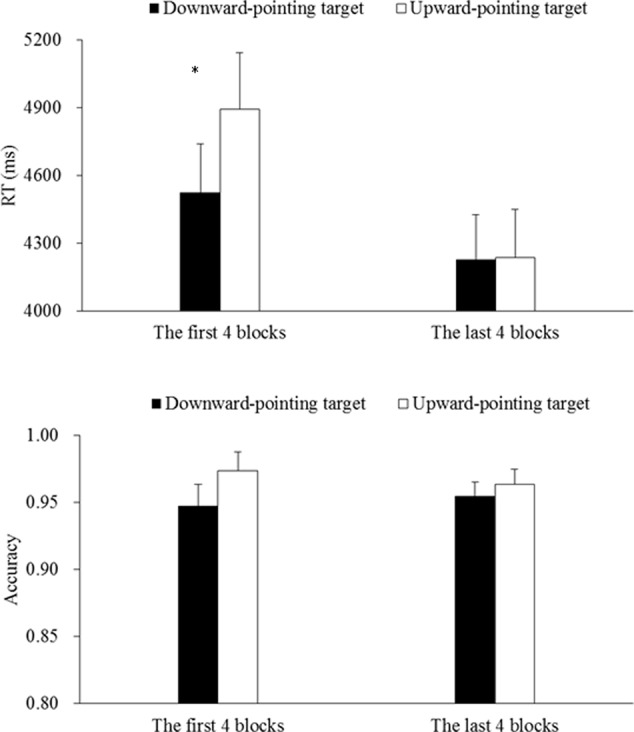
Mean RTs **(upper)** and accuracy **(lower)** for the first and last four blocks of Experiment 1. The error bars show the standard errors of means. The ^∗^ sign denotes a significant difference between two conditions when *p* < 0.05.

In this experiment, the mean accuracy of participants’ responses was high (95.9%). RT data lower than 150 ms or in excess of three standard deviation of the group mean were excluded from the subsequent data analyses, which resulted in 0.91% of the data being discarded. Preliminary analyses revealed that searching for a downward-pointing target (4282 ms, 95.2%) was comparable to searching for an upward-pointing one (4402 ms, 96.5%), both *F*s < 2.13, *p*s > 0.15. However, when we further broke down the data, we noticed that the participants’ responses speeded-up as a function of the number of experimental blocks that they had completed. Therefore, we divided the eight experimental blocks into two halves. Mean RTs calculated based on correct trials (in which the participants correctly selected the target) and accuracy data for each type of target are shown in **Figure 3**.

The 2 (Target Orientation: downward- or upward-pointing) × 2 (Block Order: the first or last 4 blocks) Analyses of Variances (ANOVAs) on the RT and accuracy data revealed a significant main effect of Block Order on the RTs, *F*(1,23) = 13.92, *p* < 0.01, $\eta_{p}^{2}$ = 0.38, but not on the accuracy data, *F*(1,23) = 0.03, *p* = 0.87. These results suggested that the participants responded more rapidly in the last four blocks (4232 ms, 95.9%) than in the first four blocks (4709 ms, 96.0%) with comparable accuracy in the two halves of the study. The results also revealed a marginally significant main effect on the RTs, *F*(1,23) = 3.14, *p* = 0.09, $\eta_{p}^{2}$ = 0.12, that was qualified by a significant interaction between Target Type and Block Order, *F*(1,23) = 5.29, *p* = 0.03, $\eta_{p}^{2}$ = 0.19. *Post hoc* pairwise comparisons revealed that in the first four blocks, searching for a downward-pointing target (4523 ms, 94.7%) was faster than searching for an upward-pointing target (4894 ms, 97.4%), *t*(23) = 2.16, *p* = 0.04, Cohen’s *d* = 0.45, with comparable accuracy, *t*(23) = 1.26, *p* = 0.22, thus indicating a significant DPTS effect of 371 ms. By contrast, in the last four blocks, searching for a downward-pointing target (4228 ms, 95.4%) was comparable to searching for an upward-pointing target (4237 ms, 96.3%), both *t*s < 1.26, *p*s > 0.22.

Considering the high (i.e., near-ceiling) accuracy in this experiment, we also log-transformed the accuracy data and performed the Target Orientation × Block Order ANOVA. None of the main or interaction effects were significant, all *F*s < 1.90, *p*s > 0.18.

Taken together, these results revealed that the DPTS effect emerged when the participants started to search for a wine bottle having a triangle in a different orientation from the others on the front label, though such an effect might be eliminated by the practice effect when the task was performed repeatedly. The emergence of the DPTS effect in the visual search for wine labels in a virtual supermarket is consistent with what has been documented in similar search performed with pictures of store shelves previously (Zhao et al., 2016), thus suggesting that wine labels, as a local packaging feature, can effectively attract consumers’ attention in visual search for a product presented on the store shelves (Thomas and Pickering, 2003; Elliot and Barth, 2012). However, the attenuation of the DPTS effect with practice was not unusual given the published visual search literature (e.g., see Hoffman et al., 1983; Hock et al., 1985; Menneer et al., 2012).

It should be noted that all 8 of the bottles within each display were presented on the top shelf on one side of the aisle in our first experiment. This is relevant given that previous research has revealed that the products presented on the top shelf receive more attention than those at the bottom (Chandon et al., 2009; see also Sunaga et al., 2016). Thus, it remains unclear whether the DPTS effect observed with the bottles presented on the top shelf would also be generalized to products that happened to be presented on other shelves. Nevertheless, we were worried that it might introduce too many confounding factors (e.g., head movement, and display size, etc.) to present the bottles on the bottom shelf in the virtual supermarket. Thus, in Experiment 2, we chose to present the eight bottles within each display on two adjacent shelves, and examine the influence of target position. What is more, we also presented the displays on both sides of the aisle to simulate a somewhat more natural shopping situation. Thus far, it remains unclear whether the orientation of the triangle on the wine labels influence consumers’ subjective ratings of and/or preference on the bottles of liquid. In order to address this issue, we also had the participants rate the stimuli after the visual search task of Experiment 2.

## Experiment 2

### Methods

Twenty-four Chinese participants (mean age = 21.04 ± 2.96 years, ranging from 18 to 28 years; 12 females and 12 males) were recruited from the same subject pool as in Experiment 1. None of the participants had taken part in Experiment 1. All aspects of the methods of this experiment were identical to those reported in Experiment 1 with the following exceptions.

**FIGURE 4:**
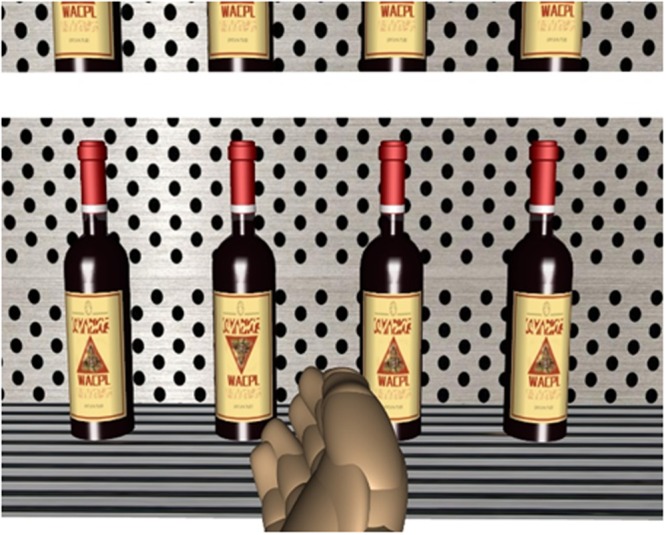
A screenshot from the right eye of the HMD for a search display (consisting of red wines as an example) when the participant was attempting to choose a target on the lower shelf in Experiment 2. The virtual hand was used to select the target.

First, in order to examine the position effect, the eight bottles of liquid were presented on two adjacent shelves (i.e., the top and second shelves), which we refer to as the upper and lower shelves, respectively. Thus, there were 4 bottles on each shelf, 0.2 m apart from each other. In order to make sure that all of the participants saw the same displays across the two adjacent shelves, the eye height in this experiment was set to 1.72 m, which was lower than that in Experiment 1. Thus, a 2 (Target Orientation: downward- or upward-pointing) × 2 (Target Location: the upper or lower shelf) within-participants experimental design was used. In order to more naturally simulate an everyday shopping experience, we also used a virtual hand to replace the blue rod in Experiment 1 (see **Figure 4** for an illustration).

Second, in this experiment, the bottles were presented on both sides of the aisle. At the beginning of each block, the participants stood at the center of the aisle, and then moved forward to respond to the displays on their right. When they had finished with all the displays on the right and arrived at the end of the aisle, they were instructed to turn around in order to be able to respond to all the displays on the other side. Therefore, the shelf displays were always on the participant’s right side in this experiment. As the participants stood at the center of the aisle, the displays were approximately 1.1 m away from where they were standing.

Third, in order to eliminate or at least reduce the practice effect observed in Experiment 1, the task in this experiment was designed to be somewhat more complicated. Specifically, the participants first had to press a button on the gamepad to identify whether the target (which had a different wine labels from all seven other bottles) was on the upper or lower shelf, then to direct the virtual hand (by turning the head) to choose the target on the selected shelf. The participant’s responses to identify the shelf where the target was located and to choose the target were both recorded. Therefore, a total of four dependent variables were used to assess the participants visual search performance, including: (1) the RTs of identification (from seeing the display to pressing a button on the game pad to indicate where the target was located on the upper or lower shelf; (2) accuracy to indicate whether the shelf where target was located was correctly identified; (3) the RTs of choosing the target, consisting of response latencies, the time durations to start moving to pointing to the target, and the time durations to press a button on the game pad to confirm the selection; and (4) accuracy to indicate whether the target was correctly chosen.

Last but by no means the least, after finishing the visual search task, all of the participants completed a survey at Unipark^4^ in order to rate the bottles (with a downward- or upward-pointing triangle in its label) presented in the visual search task as well as simple shapes of triangle (pointing downward or upward). During each trial, a picture was shown, and the participants rated the picture on the same 7-point scales as Shen et al. (2015) used, including (1) valence scale (ranging from very unpleasant to very pleasant), (2) arousal scale (ranging from very relaxing to very exciting), (3) familiarity scale (ranging from extremely unfamiliar to extremely familiar), and (4) liking scale (ranging from extreme dislike to extreme liking). At the end of the experiment, all of the participants were also asked how often they consumed and purchased alcoholic drinks in daily life (i.e., never, occasionally, sometimes, or often).

### Results

#### The DPTS Effect in the Visual Search Task

**FIGURE 5:**
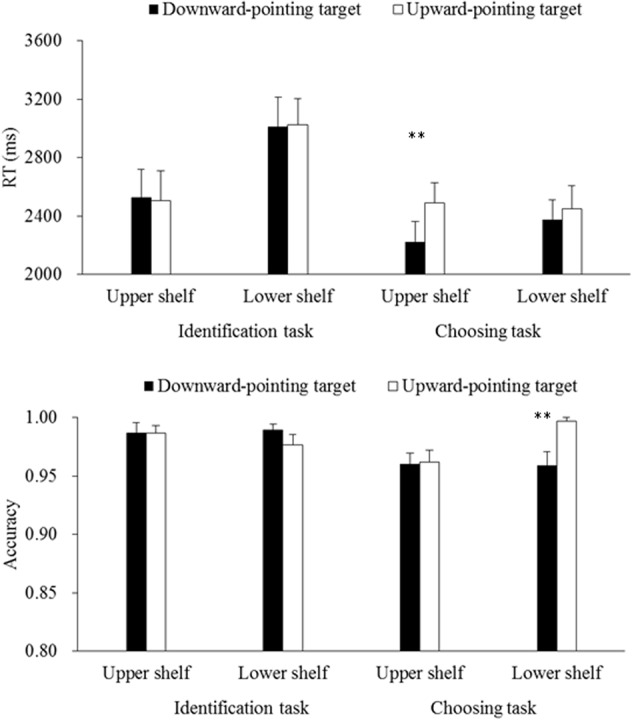
Mean RTs **(upper)** and accuracy **(lower)** for the target presented on the upper or lower shelf in Experiment 2. The error bars show the standard errors of means. The ^∗∗^ sign denotes a significant difference between two conditions when *p* < 0.01.

In this experiment, the mean accuracy of participants’ responses to identify whether the target was located on the upper or lower shelf was high (98.4% correct). After the participants had made a correct identification response, they had a high accuracy of 97.0% in responses of choosing the target. RT data (for both identification and choosing) lower than 150 ms or in excess of three standard deviation of the group means were excluded from the subsequent data analyses. This resulted in 2.7 and 2.4% of the data being discarded, respectively. Mean RTs calculated based on correct trials and accuracy data for each type of target are shown in **Figure 5**.

Considering the observed influence of practice on the DPTS effect reported in Experiment 1, a repeated-measures ANOVA – 2 (Target Orientation: downward- or upward-pointing) × 2 (Target Location: the upper or lower shelf) × 2 (Block Order: the first or last 4 blocks) was first performed on the data of Experiment 2. The results revealed a significant main effect of Block Order on the identification RTs, *F*(1,23) = 18.56, *p* < 0.001, $\eta_{p}^{2}$ = 0.45, suggesting that the participants identified the shelf where the target was located more rapidly during the last four blocks (2595 ms) than during the first four blocks (2931 ms). Nevertheless, none of other main effects of Block Order or any interaction terms between Block Order and Target Orientation/Location was significant, all *F*s < 2.93, *p*s > 0.10. Therefore, Block Order was not included in the following analyses.

Next, 2 (Target Orientation: downward- or upward-pointing) × 2 (Target Location: the upper or lower shelf) repeated-measures ANOVAs were performed on the RT and accuracy data of the identification responses. The results revealed a significant main effect of Target Location on the RTs, *F*(1,23) = 26.20, *p* < 0.001, $\eta_{p}^{2}$ = 0.53, thus suggesting that the participants identified the target more rapidly when it was presented on the upper shelf (2516 ms) than when it was presented on the lower shelf (3018 ms). None of other main or interaction effects was significant, all *F*s < 0.80, *p*s > 0.38, indicative of the absence of the DPTS effect in the identification responses.

After that, analogous analyses were performed on the RT and accuracy data of the choosing responses. The results revealed a significant main effect of Target Orientation on the RTs, *F*(1,23) = 7.00, *p* = 0.01, $\eta_{p}^{2}$ = 0.23, and on the accuracy data, *F*(1,23) = 5.25, *p* = 0.03, $\eta_{p}^{2}$ = 0.19. However, these main effects were qualified by the marginally significant interaction terms between Target Orientation and Target Location on the RTs, *F*(1,23) = 3.90, *p* = 0.06, $\eta_{p}^{2}$ = 0.15, and on the accuracy data, *F*(1,23) = 4.01, *p* = 0.06, $\eta_{p}^{2}$ = 0.15. What is more, there was also a significant main effect of Target Location on the accuracy data, *F*(1,23) = 4.26, *p* = 0.05, $\eta_{p}^{2}$ = 0.16, but not on the RTs, *F*(1,23) = 0.60, *p* = 0.45. In order to interpret these interaction terms, the data were further broken down and pairwise comparisons were performed for each target location. The results revealed that choosing a downward-pointing target on the upper shelf (2223 ms, 96.1%) was faster than choosing an upward-pointing target on the same shelf (2492 ms, 96.3%), *t*(23) = 3.68, *p* < 0.01, Cohen’s *d* = 0.76, with comparable accuracy, *t*(23) = 0.13, *p* = 0.90, indicative of a significant DPTS effect of 269 ms. By contrast, a downward-pointing target was chosen less accurately on the lower shelf (2376 ms, 96.0%) than an upward-pointing target on the same shelf (2453 ms, 99.7%), *t*(23) = 3.21, *p* < 0.01, Cohen’s *d* = 0.18, with comparable RTs, *t*(23) = 0.87, *p* = 0.40, indicative of the absence of the DPTS effect.

Similar to Experiment 1, we once again log-transformed the accuracy data in Experiment 2, and performed the Target Orientation × Target Location ANOVAs. None of the main or interaction effects were significant on the transformed accuracy data of the identification responses, all *F*s < 1.90, *p*s > 0.18. As for the transformed accuracy data of choosing responses, the results revealed a significant main effect of Target Orientation, *F*(1,23) = 5.10, *p* = 0.03, $\eta_{p}^{2}$ = 0.18, a marginally significant main effect of Target Location, *F*(1,23) = 3.70, *p* = 0.07, $\eta_{p}^{2}$ = 0.14, and a marginally significant interaction term, *F*(1,23) = 4.03, *p* = 0.06, $\eta_{p}^{2}$ = 0.15. Therefore, the patterns of results we obtained with transformed accuracy data were consistent with those with raw data.

#### Ratings of Stimuli in the Downward- and Upward-Pointing Orientations

The mean ratings for each stimulus in the downward- and upward-pointing orientations were summarized (see **Table 1**). The 2 (Triangle Orientation: downward- or upward-pointing) × 4 (Stimulus Type, red wine, white wine, whiskey, or baijiu) ANOVAs on these scores revealed a significant main effect of Triangle Orientation on familiarity scores, *F*(1,23) = 4.26, *p* = 0.05, $\eta_{p}^{2}$ = 0.16, but not on any of the other three scores, all *F*s < 2.06, *p*s > 0.16. These results therefore suggest that the same bottles were rated as more familiar when the triangle in its label was oriented in the downward-pointing direction than when they were shown in the upward-pointing orientation instead, with comparable ratings of pleasantness, arousal, and liking scores. The results also revealed significant main effects of Stimulus Type on valence, *F*(1,23) = 5.16, *p* < 0.01, $\eta_{p}^{2}$ = 0.18, familiarity, *F*(1,23) = 4.93, *p* < 0.01, $\eta_{p}^{2}$ = 0.18, and liking scores, *F*(1,23) = 10.80, *p* < 0.001, $\eta_{p}^{2}$ = 0.32. None of other main effects or interaction terms was significant on any of the scores, all *F*s < 1.67, *p*s > 0.18. By contrast, the simple triangle, when presented by itself, received lower valence scores when oriented in the downward-pointing direction than when it was presented in the upward-pointing orientation instead, *F*(1,23) = 4.18, *p* = 0.05, $\eta_{p}^{2}$ = 0.15, with comparable arousal, familiarity, and liking scores, all *F*s < 2.83, *p*s > 0.10. Taken together, these results therefore suggest that the downward-pointing triangle was considered to be less pleasant than the same stimulus oriented in the upward direction, whereas such ratings did not influence how pleasant people considered the bottles having these triangles on the labels.

**Table 1:** Ratings of valence, arousal levels, familiarity, and liking (on 7-point scales) for downward- and upward-pointing stimuli (with SDs in parentheses) in Experiment 2.

Ratings	Orientation	Stimuli				
		Triangle	Red wine	White wine	Whiskey	Chinese baijiu
Valence	Downward-pointing	3.50 (0.31)	4.71 (0.23)	4.46 (0.31)	3.71 (0.29)	3.54 (0.31)
	Upward-pointing	4.13 (0.21)	4.75 (0.24)	4.58 (0.22)	4.08 (0.26)	3.67 (0.34)
Arousal	Downward-pointing	4.21 (0.28)	4.71 (0.22)	4.17 (0.31)	3.88 (0.33)	4.58 (0.27)
	Upward-pointing	4.13 (0.27)	4.67 (0.20)	4.17 (0.25)	4.21 (0.28)	4.50 (0.32)
Liking	Downward-pointing	5.71 (0.21)	5.25 (0.24)	4.38 (0.36)	4.25 (0.33)	4.96 (0.29)
	Upward-pointing	5.79 (0.20)	4.79 (0.29)	4.25 (0.30)	3.75 (0.33)	4.79 (0.35)
Familiarity	Downward-pointing	3.83 (0.26)	4.67 (0.25)	4.33 (0.27)	3.63 (0.29)	3.29 (0.24)
	Upward-pointing	4.25 (0.17)	4.75 (0.21)	4.50 (0.21)	4.08 (0.22)	3.21 (0.28)

Last, but by no means the least, the participants reported that they occasionally (83%) or never (17%) consumed alcoholic drinks in daily life, and they occasionally (46%) or never (54%) purchased alcoholic drinks on their own.

### Discussion

The results of Experiment 2 revealed a significant DPTS effect when the participants chose the target bottle on the top shelf, whereas no such effect was observed with the target bottle presented on the second shelf or in their responses of identifying the shelf on which the target was located. Taken together with the results of Experiment 1, we replicated the DPTS effect with the visual search for bottles of wine in a virtual shopping situation, thus suggesting that incorporating a downward-pointing triangle on a wine label might influence people’s attention in situations that are more complex and realistic than laboratory-based experiments.

On the other hand, even though the participants considered the downward-pointing triangle to be less pleasant than the upward-pointing one (see also Shen et al., 2015), we found no significant difference in the pleasantness rating scores of the same bottles of wine with a triangle pointing downward vs. upward. It is worth noting that these results are inconsistent with Westerman et al.’s (2013) findings that bottles of water or vodka with several downward-pointing triangles on their labels were rated as being less liked, less appealing, and less likely to be purchased than those with upward-pointing ones. It should be noted that their study and ours used different types of drinks, different label designs, and different groups of participants, all of which might contribute to the discrepancy between the rating results (see also Spence, 2012). Most importantly, several small triangles were presented on the left or right of the label in their study, which makes the triangles a more salient feature of the packaging, whereas we only presented one distinctive triangle on the center of the wine labels. Taken together, these results also suggest that people’s ratings of the bottles with triangles on the labels might be modulated by many other factors, such as the contextual information.

## General Discussion

In the present study, we ran two VR-based experiments to examine the influence of having triangles (downward or upward oriented) on wine labels on people’s visual search for wines. The participants walked along the wine aisle in a virtual supermarket and searched for the wine bottle on the shelf that had a different triangle on its label from the other bottles. Generally speaking, the results of both experiments revealed that, if anything, choosing a bottle with a downward-pointing triangle on its label was faster than when it had an upward-pointing triangle on the label instead. It should be noted that these results are consistent with Zhao et al.’s (2016) findings with images of store shelves. Once again, we replicate the DPTS effect with naturalistic stimuli where the triangle was only a local feature in a simulated shopping situation.

These results cannot easily be interpreted by the “Shape of Threat” account proposed by Larson et al. (2007, see also Larson et al., 2012; Watson et al., 2012), for at least two reasons. For one, the bottles of wine with downward-pointing triangles on their labels were not rated as being any more unpleasant than those with upward-pointing triangles. Due to the lack of the associations between the bottles of wines with a downward-pointing triangle in their labels and negative subjective emotional ratings, the facilitated search for these bottles of wines may not be simply attributed to the affective features of the stimuli. For another, these wine bottles that the participants were asked to choose in a virtual store has no relation to threat of any kind whatsoever. Alternatively, the DPTS effect observed with non-threatening images (Shen et al., 2015; Zhao et al., 2016) and objects, as in the present study, are more in line with the possibility that the downward-pointing triangles more readily capture people’s visual attention because of particular perceptual features, such as the lack of stability and people’s expectations of the consequences of that perceived instability. Previous studies have shown that unstable shapes might be ascribed feelings of fear (Pavlova et al., 2005), while unstable-looking logos might be used to infer the presence of unsafe conditions (Rahinel and Nelson, 2016).

The results of the present study also revealed a position effect in visual search for the wines. On the one hand, the participants were faster to identify the target when it was presented on the top shelf than when it was presented on the second shelf. On the other hand, the position of the target shelf also modulated the DPTS effect. These results are in line with other position effects whereby, for instance, products presented at a higher location in the rack tend to attract more attention (Chandon et al., 2009; though see also Sunaga et al., 2016). What is more, it should be noted that consumers might use in-store displays as a source of information when trying to assess the quality and likely price of the products (Valenzuela and Raghubir, 2009). Here it is worth noting that products on higher shelves are generally assumed to be of better quality and have higher price than those placed on lower shelves (Valenzuela et al., 2013; Valenzuela and Raghubir, 2015). Therefore, in the present study, wines presented on the top shelf might attract more attention and are considered to be better, which might be important factors for the DPTS effect to emerge. It is worth noting that the vertical location of an object might also be associated with stimulus valence (for a recent review, see Cian, 2016). For example, negative words are recognized more rapidly when placed at the bottom of a computer screen, whereas positive words are recognized more rapidly when they placed at the top of the screen, suggesting an association between “being higher” and “being better” (Meier and Robinson, 2004). Taken together, these results suggest that having the downward-pointing triangle on the label of products which are assumed to be good might guide people’s attention more efficiently, whereas it might not work if the products are not considered in this way.

There are also some straightforward limitations in the present study that should be acknowledged. First, as for a lab experiment conducted on a university campus, the participants were Chinese college students (see Henrich et al., 2010, for the discussion of biased samples) who at most, only occasionally drink or purchase alcoholic drinks. Previous research has revealed that older frequent wine consumers’ evaluation of wine was more strongly influenced by brand and packaging (Mueller and Szolnoki, 2010), while the present study conducted with young inexperienced consumers might even underestimate the influence of wine labels on consumers’ visual attention. What is more, it will be also interesting in future research to examine the possible cross-cultural differences in the participants’ ratings of the stimuli. On the other hand, it is also worth bearing in mind that those customers who purchase alcoholic drinks in stores are not necessarily the ones who drink it. For example, back in the 1950’s, Cheskin (1957, see also Cheskin, 1981) reported that rounding the corners of the labels on the front of one brand of gin bottle made these more appealing to these female customers who purchase gins for their husbands to drink.

Second, in addition to the triangles on the wine labels shown in the present study, each bottle also has an upward-pointing V form in its shape because the neck of the bottle is narrower than the body, which might introduce an additional confounding factor of “global/local congruency.” That is, when a bottle has a downward-pointing triangle on its label, the orientations of the triangle on the wine label and the bottle are inconsistent with each other; whereas the orientation of the upward-pointing triangle on the wine label is consistent with the bottle. Therefore, future research is called for in which the shape of the product is better controlled for. What is more, it will be also interesting to use heterogeneous distractors in other shapes on the wine labels such as circles and squares, which might be a more natural situation in daily life.

## Conclusion

Once again, our results demonstrate how the appearance of the bottles, in particular, the design of the wine labels, might guide consumers’ attention when searching for the product presented on the store shelves (Thomas and Pickering, 2003; Elliot and Barth, 2012; Zhao et al., 2016). Changing the labels of the product might be a more efficient and effective way to attract consumers’ attention than changing the appearance of the product, while increased attention might lead to greater likelihood of it being chosen (Orquin and Loose, 2013).

## Ethics Statement

This study was carried out in accordance with the ethical standards for conducting research established by the American Psychological Association with written informed consent from all subjects. The protocol was approved by the ethics committee of the Psychology Department of Tsinghua University.

## Author Contributions

Each of the listing co-authors made the following contributions to the paper: HZ, FH, CS, and XW co-developed the idea for the study. HZ, FH, and XW collaboratively designed the study. HZ collected the data and conducted the data analysis. HZ, FH, CS, and XW conducted the interpretation of the data, and drafted the manuscript. All of the authors have read and approved the final version of the manuscript.

## Conflict of Interest Statement

The authors declare that the research was conducted in the absence of any commercial or financial relationships that could be construed as a potential conflict of interest. The reviewer AC and handling Editor declared their shared affiliation.

## Footnotes

1 Here it is worth noting that many wine producers also use the weight of the bottle to try and convey product quality too (see Piqueras-Fiszman and Spence, 2012).

2 Zhao et al. (2016) used a within-participants design with a sample of 20 participants in their Experiment 1, and documented a significant DPTS effect of 164 ms (*SD* = 131 ms) with triangles on wine labels. While we set the Type I error probability of the present study at 0.05, we need at least nine participants (resulting in nine pairs of data) to obtain a power of 0.9 for the present study.

3 Note that baijiu is a traditional Chinese distilled alcohol which has a clear appearance. It is often referred to as “Chinese liquor/spirits” in English or “white wine” in Chinese (see Wan et al., 2015).

4 www.unipark.de
